# Arsenic Resistance and Prevalence of Arsenic Resistance Genes in *Campylobacter jejuni* and *Campylobacter coli* Isolated from Retail Meats 

**DOI:** 10.3390/ijerph10083453

**Published:** 2013-08-07

**Authors:** Aneesa Noormohamed, Mohamed K. Fakhr

**Affiliations:** Department of Biological Science, The University of Tulsa, Tulsa, OK 74104, USA; E-Mail: aneesa-noormohamed@utulsa.edu

**Keywords:** *Campylobacter*, arsenic resistance, *arsP*, *arsR*, *arsC*, *acr3*, *arsB*, arsenic operon, retail meats

## Abstract

Studies that investigate arsenic resistance in the foodborne bacterium *Campylobacter* are limited. A total of 552 *Campylobacter* isolates (281 *Campylobacter jejuni* and 271 *Campylobacter coli*) isolated from retail meat samples were subjected to arsenic resistance profiling using the following arsenic compounds: arsanilic acid (4–2,048 μg/mL), roxarsone (4–2048 μg/mL), arsenate (16–8,192 μg/mL) and arsenite (4–2,048 μg/mL). A total of 223 of these isolates (114 *Campylobacter jejuni* and 109 *Campylobacter coli*) were further analyzed for the presence of five arsenic resistance genes (*arsP*, *arsR*, *arsC*, *acr3*, and *arsB*) by PCR. Most of the 552 *Campylobacter* isolates were able to survive at higher concentrations of arsanilic acid (512–2,048 μg/mL), roxarsone (512–2,048 μg/mL), and arsenate (128–1,024 μg/mL), but at lower concentrations for arsenite (4–16 μg/mL). Ninety seven percent of the isolates tested by PCR showed the presence of *arsP* and *arsR* genes. While 95% of the *Campylobacter coli* isolates contained a larger arsenic resistance operon that has all of the four genes (*arsP*, *arsR*, *arsC* and *acr3*), 85% of the *Campylobacter jejuni* isolates carried the short operon (*arsP*, and *arsR*). The presence of *arsC* and *acr3* did not significantly increase arsenic resistance with the exception of conferring resistance to higher concentrations of arsenate to some *Campylobacter* isolates. *arsB* was prevalent in 98% of the tested *Campylobacter jejuni* isolates, regardless of the presence or absence of *arsC* and *acr3*, but was completely absent in *Campylobacter coli*. To our knowledge, this is the first study to determine arsenic resistance and the prevalence of arsenic resistance genes in such a large number of *Campylobacter* isolates.

## 1. Introduction

Recent studies reporting the presence of arsenic residues in chicken [1,2,3] have led to the voluntary stoppage of the sale of the most commonly used orgnoarsenical, roxarsone, produced by Alpharma, a subsidiary of Pfizer Inc. [4]. Resistance to arsenic has been linked to the use of arsenical compounds in the feed of poultry for pigmentation [5], growth promotion and weight gain [6,7,8] and for the treatment of parasites [5,7]. A recent study suggested that the role of roxarsone on growth promotion in chicken is through modifying the expression levels of some cell growth and energy metabolism associated genes [9]. Poultry manure contaminated with arsenic can contribute to an increase in the concentration of arsenic in the environment [10]. 

Although not as extensively studied as antimicrobial resistance, arsenic resistance in *Campylobacter* is beginning to garner attention. Few years ago, an arsenic resistance operon was identified in *Campylobacter jejuni* [11]. Another study has also shown the presence of a four-gene arsenic resistance operon in *Campylobacter lari* [12]. More recently, Shen *et al.* discussed the contribution of *arsB*, a putative efflux transporter gene that is not part of the previously characterized operon, to arsenic resistance in *Campylobacter jejuni* [13]. Including the recent *arsB* study above, only three studies are currently available in which *Campylobacter* isolates mostly from retail poultry were screened for their arsenic resistances [11,13,14]. This might be due to the lack of data on the amounts of these substances in the feed or its impact on human health [6]. 

Although the arsenic resistance operon is found mostly in Gram negative organisms, it has been found in Gram positive organisms as well, and is thought to originate from Gram positive bacteria [15,16]. The arsenic resistance determinants can be found in the chromosome as in the case of *Escherichia coli* or on transmissible plasmids as in the case of *Staphylococcus aureus* [15,17,18]. Generally, arsenic resistance operons contain three to five genes [15,16]. The 3-gene system found on *E. coli* and *P. aeruginosa* chromosomes and on *S. aureus* plasmids contains *arsR* (transcriptional repressor), *arsB* (arsenite permease), and *arsC* (arsenate reductase) [16,17,18]. The 5-gene system found on *E. coli* plasmids and *A. multivorum* plasmids contains *arsR* (arsenite inducible repressor), *arsD* (negative regulatory protein), *arsA* (ATPase), *arsB* (asenite efflux pump), and *arsC* (arsenate reductase) which converts As (V) to As (III) [16]. A 4-gene system is also available such as the one found in *Campylobacter jejuni*, *Campylobacter lari*, *B. subtilis*, *S. marsecens*, and *T. ferrooxidans* [11,12,16,19,20]. The *Campylobacter jejuni* arsenic resistance operon has been sequenced and shown to contain two to four genes [11]. The four genes that have been identified are *arsR* (a transcriptional repressor), *arsP* (a putative membrane permease), *arsC* (an arsenate reductase), and *acr3* (an efflux protein). 

The aim of the present study was to determine the arsenic resistance and the prevalence of arsenic resistance genes in *Campylobacter coli* and *Campylobacter jejuni* isolated from retail meats purchased from the Tulsa (OK, USA) area grocery stores. To our knowledge, this is the first study to determine the prevalence of these genes in such a large number of isolates. 

## 2. Experimental Section

### 2.1. Bacterial Isolates

A total of 552 *Campylobacter* isolates (281 *Campylobacter jejuni* and 271 *Campylobacter coli*) previously isolated from retail meat samples in our laboratory [21,22] were used for arsenic resistance profiling in this study. The isolates were from chicken (*n =* 130), turkey (*n =* 19), pork (*n =* 6), beef livers (*n =* 102), chicken livers (*n =* 272), and chicken gizzards (*n =* 23). All isolates were kept frozen at −80 °C in Brucella broth (Becton Dickinson, Sparks, MD, USA) with 20% glycerol. A subset of 223 *Campylobacter* isolates out of the above mentioned 552 isolates was used for screening for the presence or absence of the tested arsenic resistance genes. The 223 *Campylobacter* isolates were representing one isolate out of each positive meat sample [21,22] and their meat sources were as follow: chicken (*n =* 49), turkey (*n =* 7), pork (*n =* 2), beef livers (*n =* 39), chicken livers (*n =* 113), and chicken gizzards (*n =* 13). 

### 2.2. Arsenic Resistance Screening

The agar dilution plate method was used to screen for arsenic resisatnce. Isolates were grown on Mueller-Hinton (MH) agar (Difco, Sparks, MD, USA) supplemented with 5% laked horse blood (Hemostat Laboratories, Dixon, CA, USA) and incubated for 48 h at 42 °C at microaerophilic conditions. Cultures were then added to Mueller-Hinton broth (Difco), adjusted to turbidity equal to a 0.5 McFarland standard, and inoculated onto 6-inch MH agar plates supplemented with 5% defibrinated sheep blood and the arsenic compound at different concentrations (Table 1) using a 96-point replicator starting at the lowest concentration of each antimicrobial and working up. Since there are no published breakpoints for the arsenical compounds, we used the two published studies as a guide [6,11] and we increased the range to ten different concentrations for each of the four tested arsenic compounds in our study (Table 1). The plates were incubated at 42 °C for 48 h under microaerophilic conditions. The plates were then read for growth or no growth and denoted as resistant or susceptible to this specific arsenic concentration, respectively.

**Table 1:** The arsenical compounds and their concentrations used for susceptibility testing.

Arsenicals	Conc. 1 (µg/mL)	Conc. 2 (µg/mL)	Conc. 3 (µg/mL)	Conc. 4 (µg/mL)	Conc. 5 (µg/mL)	Conc. 6 (µg/mL)	Conc. 7 (µg/mL)	Conc. 8 (µg/mL)	Conc. 9 (µg/mL)	Conc. 10 (µg/mL)
**Arsanilic Acid**	4	8	16	32	64	128	256	512	1,024	2,048
**Arsenate**	16	32	64	128	256	512	1,024	2,048	4,096	8,192
**Arsenite**	4	8	16	32	64	128	256	512	1,024	2,048
**Roxarsone**	4	8	16	32	64	128	256	512	1,024	2,048

### 2.3. DNA Extraction

Bacterial DNA extracts used in polymerase chain reaction (PCR) were prepared from *Campylobacter* cultures using the single-cell lysing buffer (SCLB) method [23]. Isolates were struck to Mueller-Hinton agar (MH; Difco), and incubated at 42 °C for 48 h under microaerophilic conditions. One colony was picked from the plate and suspended in 40 µL of SCLB solution in a 0.2 mL microtube. The SCLB solution contained 10 µL of 5 mg/mL proteinase K (Amresco, Solon, OH, USA) and 1.0 mL of TE buffer (10 mM Tris-HCl (J. T. Baker, Phillipsburg, NJ, USA) and 1 mM EDTA (Fisher, Fair Lawn, NJ, USA). The cells were lysed by heating at 80 °C for 10 min, followed by cooling to 55 °C for 10 min, using a thermocycler (Eppendorf, Hamburg, Germany). The suspension was diluted 1:2 in sterile double distilled water and centrifuged in a microfuge at 14,000 rpm for 30 s to remove cellular debris. The supernatant was used as DNA template for PCR.

### 2.4. PCR for Arsenic Genes

**Figure 1:**
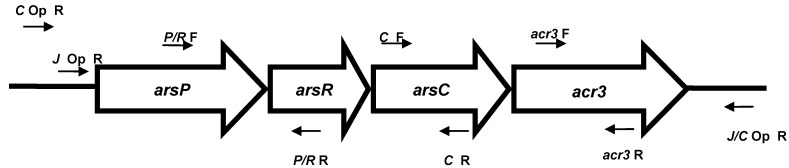
A schematic diagram of the arsenic operon genes and the location of the PCR primers used for amplification. *C* Op F (*Campylobacter coli* operon forward primer), *J* Op F (*Campylobacter* *jejuni* operon forward primer), J/C Op R (Both species operon reverse primer), *P/R* F (*arsP/arsR* forward primer), *P/R* R (*arsP/arsR* reverse primer), *C* F (*arsC* forward primer), *C* R (*arsC* reverse primer), *acr* F (*acr3* forward primer), and *acr3* R (*acr3* reverse primer).

The selected arsenic resistant isolates were tested for the presence of arsenic resistance genes and operons by a regular PCR reaction using the primers designed specifically for *Campylobacter* *jejuni* and *Campylobacter coli* (Figure 1). For *arsC*, the following primer pair sequence [11] was used, Forward 5’-ATGAAACTTGCATTTATTTGTATT-3’, Reverse 5’-CTAACATGTAAAGTCCTTAAGAGAA-3’. For *acr3*, the following primer pair [11] was used, Forward 5’-ATGTTAGGTTTTATCGATAGAT-3’, Reverse 5’-TCATGAGGCTTGATTCATTTTT-3’. Due to some variations in the DNA sequence particularly for *arsR* between *Campylobacter jejuni and Campylobacter coli*, the following newly designed PCR primer pair in this study was used to detect simultaneously the presence of both *arsP* and *arsR* genes in isolates of the two species, Forward 5’-AAAGCTATATTTTTAGGTGCTTTAACG-3’, Reverse 5’-TAAATGTCTTGAAAGTCTTG-3’. To amplify the whole operon even in case of missing *arsC* and *acr3*, the following primers were used, Forward for *Campylobacter jejuni* 5’-CATTCATTTTAGAGTTATTGCGTATAAAACATACT-3’ [11], Forward for *Campylobacter coli* 5’-TAAATAGCACTACTTAAACAACAAATAGCCAAAC-3’ (Designed in this study), Reverse for both *Campylobacter jejuni* and *Campylobacter coli* 5’-ATACCCAAAGAGCAAAGTTTTATATAATGTTTGA-3’ (Designed in this study). For the *arsB*, the following newly published primer pair [13] was used to screen both species, Forward 5’-GGAATTTACCTATTTGGGTAT-3’, Reverse 5’-ATATTAATGCCTTTTCTAGCC-3’. A schematic presentation of the arsenic operon genes and the location of their PCR primers are shown in Figure 1. The PCR was carried out in 25 µL reactions. Each 25 µL reaction contained 12.5 µL *Taq* Polymerase Master Mix (Qiagen Inc., Valencia, CA, USA), 3.5 µL sterile water (Qiagen), 1 µL (25 pmol) each primer (IDT, Coralville, IA, USA), and 3 µL of template DNA. The cycling conditions were set as follows for the arsenic genes tested: 95 °C for 5 min, 95 °C for 1 min, 50 °C for 1 min, 72 °C for 1 min, and 72 °C for 10 min. Steps 2 through 4 were repeated for 30 cycles. For the arsenic operon amplification, the extension time was increased to 3 min instead of 1 minute for each of the 30 cycle. Once the cycles were complete, reactions were held at 4 °C until gel electrophoresis. Ten microliters of PCR product was subjected to horizontal electrophoresis in a 1% agarose gel in 1 X Tris-acetate-EDTA (TAE) buffer. A 1 kb plus ladder (Bioneer, Alameda, CA, USA) was used as the molecular marker. Gels were viewed and recorded by ultraviolet transillumination, using a UV imager. The expected amplicon sizes were 945 bp for *arsP/R*, 423 bp for *arsC*, 1044 bp for *acr3*, and 1130 bp for *arsB*. Sterile water was used as the negative control. The DNA of few amplicons was sequenced in house to assure specificity.

## 3. Results and Discussion

### 3.1. Arsenic Resistance Screening

Studies on arsenic resistance in *Campylobacter* species are limited in the literature [6,11,12]. In this study, a total of 552 *Campylobacter* isolates (281 *Campylobacter jejuni* and 271 *Campylobacter coli*) isolated from retail meat samples were subjected to arsenic resistance profiling using the agar dilution method to the following arsenic compounds: arsanilic acid (4–2,048 μg/mL), roxarsone (4–2,048 μg/mL), arsenate (16–8,192 μg/mL) and arsenite (4–2,048 μg/mL). The 10 different concentrations tested for each arsenic compound is listed in details in Table 1. The isolates were from chicken (*n =* 130), turkey (*n =* 19), pork (*n =* 6), beef livers (*n =* 102), chicken livers (*n =* 272), and chicken gizzards (*n =* 23). As shown in Table 2, most of the 552 *Campylobacter* isolates were able to survive at higher concentrations of arsanilic acid (512–2,048 μg/mL), roxarsone (512–2,048 μg/mL), medium concentrations of arsenate (128–1,024 μg/mL), but at lower concentrations for arsenite (4–16 μg/mL). It is apparent from the results above that the *Campylobacter* isolates screened in this study have a higher resistance to the organoarsenicals that is known to be used in production animal feeds (arsanilic acid and roxarsone). Using these compounds in feed can cause a selective pressure towards more resistant *Campylobacter* isolates. The isolates were not highly resistant to arsenite which is the most toxic by-product of the organoarsenicals. The high rates of resistance observed here are of some concern since it might be an indication of an extensive use of arsenic in the feed and raises a concern about the levels of this heavy metal in the retail meat consumed. The concern that retail meat products accumulate enough arsenical compounds that they may become carcinogenic and potentially affecting human health cannot be eliminated [14]. We hope that the voluntary stoppage of the sale of roxarsone by Alpharma will decrease such a risk to human health [4]. It is worth mentioning that our meat samples were collected before this voluntary stoppage of the sale of roxarsone. 

**Figure 2:**
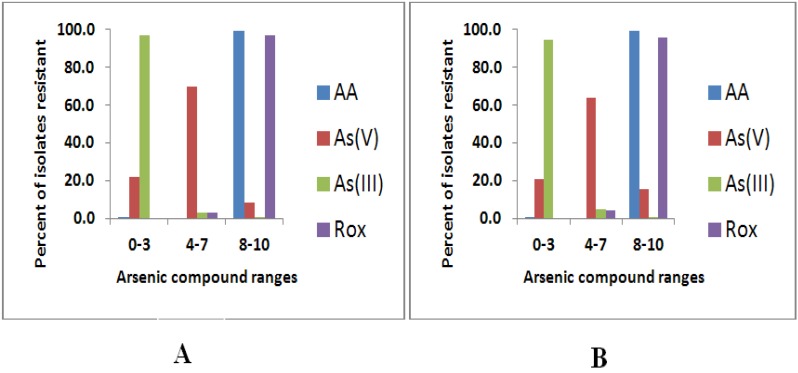
Percentage of arsenic resistance of 281 *Campylobacter jejuni* (**A**) and 271 *Campylobacter coli* isolates (**B**) to ten concentrations of the four arsenic compounds tested. Please refer to Table 1 for the detailed concentrations from 0 to 10.

Arsenic resistance to the four tested arsenic compounds did not vary much between the tested 281 *Campylobacter jejuni* and 271 *Campylobacter coli* isolates (Table 2, Figure 2). The only noticeable difference was that 15.5% of the *Campylobacter coli* isolates were resistant to higher concentrations of arsenate (2,048–8,192 μg/mL) where only 8.5% of *Campylobacter jejuni* isolates were resistant to the same concentration range.

**Table 2:** Percentage of Arsenic resistance of 281 *Campylobacter jejuni* and 271 *Campylobacter coli* isolates to ten concentrations of the four arsenic compounds tested. Please refer to Table 1 for the detailed concentrations from 0 to 10.

	*Campylobacter coli*				* Campylobacter jejuni*			
**Conc**.	AA (%)	As(V) (%)	As(III) (%)	Rox (%)	AA (%)	As(V) (%)	As(III) (%)	Rox (%)
**0**–**3**	1/271 (0.4)	56/271 (20.7)	256/271 (94.5)	0/271 (0.0)	1/281 (0.4)	61/281 (21.7)	272/281 (96.8)	0/281 (0.0)
**4**–**7**	0/271 (0.0)	173/271 (63.8)	13/271 (4.8)	12/271 (4.4)	0/281 (0.0)	196/281 (69.8)	8/281 (2.8)	9/281 (3.2)
**8**–**10**	270/271 (99.6)	42/271 (15.5)	2/271 (0.7)	259/271 (95.6)	280/281 (99.6)	24/281 (8.5)	1/281 (0.4)	272/281 (96.8)

### 3.2. Arsenic Resistance Gene Screening

A subset of 223 *Campylobacter* isolates (114 *Campylobacter jejuni* and 109 *Campylobacter coli*) were selected and further analyzed for the presence or absence of five arsenic resistance genes (*arsP*, *arsR*, *arsC*, *acr3, and arsB*) by PCR. The 223 *Campylobacter* isolates were selected to represent one isolate out of each positive retail meat sample. Ninety seven percent (217/223) of the isolates tested by PCR showed the presence of *arsR* and *arsP* genes (Table 3). While 95% (104/109) of the *Campylobacter coli* isolates contained a larger arsenic resistance operon that has all of the four genes (*arsP*, *arsR*, *arsC* and acr3), only 15% (17/114) of the *Campylobacter jejuni* isolates carried the large operon (Table 3). In a previous study, the arsenic resistance operon was characterized in *Campylobacter jejuni* [11]. It was found to contain *arsP*, *arsR*, *arsC* and *acr3* [11]. In some isolates though, the authors of the study found that only *arsR* and *arsP* were detected and not the *arsC* and *acr3* [11]. A more recent study that investigated the distribution of colonization and antimicrobial resistance genes in *Campylobacter jejuni* isolated from chicken reported 90% prevalence of *arsR* and47% prevalence of *arsC* [24]. 

**Table 3:** Percentage of arsenic resistance genes present in 114 *Campylobacter jejuni* and 109 *Campylobacter coli* isolates.

Genes	*Campylobacter jejuni*		*Campylobacter coli*	
	Isolates	%	Isolates	%
*arsR*	108/114	94.7	109/109	100
*arsP*	108/114	94.7	109/109	100
*arsC*	17/114	14.9	105/109	96.3
*acr3*	20/114	17.5	104/109	95.4
*arsB*	112/114	98	0/109	0

Ninety eight percent (112/114) of the tested *Campylobacter jejuni* isolates showed the presence of *arsB*, while none of the 109 *Campylobacter coli* contained the gene (Table 3). Interestingly enough, the only two *Campylobacter jejuni* isolates that did not harbor *arsB* were also lacking *arsC* and *acr3* and were resistant to roxarsone, arsenate, and arsanilic acid (data not shown). While these are only two isolates but the data might suggest that there is still another unexplored mechanism of arsenic resistance in *Campylobacter* other than *arsB*. One cannot completely rule out the possibility of the involvement of the cmeABC efflux pump in this regard but this needs to be verified by mutagenesis experiments. Shen *et al.* (2013) reported a broad distribution of *arsB* and *acr3* in *Campylobacter jejuni* isolates of different sources and suggested that at least one of these two genes is required for adaptation of *Campylobacter* to arsenic rich niches [13]. The complete absence of *arsB* from our screened *Campylobacter coli* isolates and its presence in 98% of the *Campylobacter jejuni* isolates, even those harboring acr3, highlight the possibility of the presence of another mechanism in *Campylobacter* arsenic resistance that is yet to be characterized.

**Figure 3:**
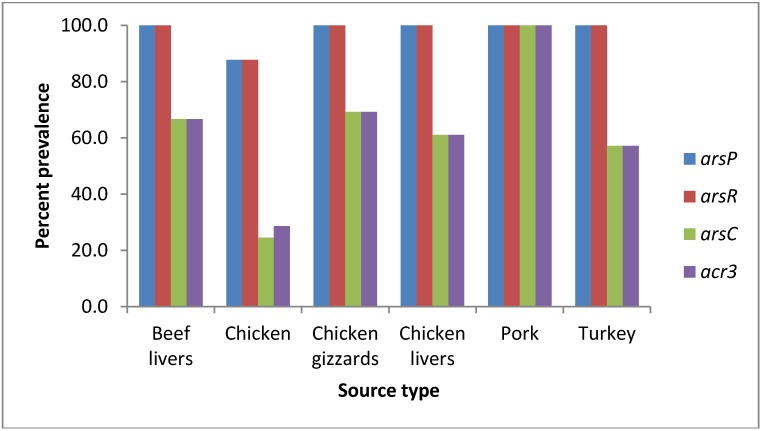
A histogram showing the percentage prevalence of arsenic resistance genes in 223 *Campylobacter* screened isolates according to the meat source type of these isolates.

As shown in Table 4, the presence of *arsC* and *acr3* (large operon) appears to be more prevalent in *Campylobacter coli* than *Campylobacter jejuni* isolates rather than in a particular meat source. Within the *Campylobacter jejuni* isolates the prevalence of *arsC* and *acr3* was higher in turkey 60% (3/5), and chicken gizzards 60% (6/10) isolates (Table 4) compared to the average prevalence of these two genes (14.9% and 17.5%) from all meat sources tested (Table 3). As shown in Figure 3 and Table 4 and if not taking the species into consideration, the prevalence of *arsC* and *acr3* (large operon) appears to be higher in the pork isolates (100%) followed by chicken gizzards (69%), beef livers (67%), chicken livers (61%), turkey (57%), and finally chicken (29%).

**Table 4:** Percentage of arsenic resistance genes present in 114 *Campylobacter jejuni* and 109 *Campylobacter coli* isolates according to the isolates meat source.

Source	*arsP* *np/n (%)			*arsR* *np/n (%)			*arsC* *np/n (%)			*acr3* *np/n (%)		
	*C. jejuni*	*C. coli*	Total	*C. jejuni*	*C. coli*	Total	*C. jejuni*	*C. coli*	Total	*C. jejuni*	*C. coli*	Total
**Beef livers**	13/13 (100)	26/26 (100)	39/39 (100)	13/13 (100)	26/26 (100)	39/39 (100)	0/13 (0)	26/26 (100)	26/39 (66.7)	0/13 (0)	26/26 (100)	26/39 (66.7)
**Chicken**	34/40 (85)	9/9 (100)	43/49 (87.8)	34/40 (85)	9/9 (100)	43/49 (87.8)	3/40 (7.5)	9/9 (100)	12/49 (24.5)	6/40 (15)	8/9 (88.9)	14/49 (28.6)
**Chicken gizzards**	10/10 (100)	3/3 (100)	13/13 (100)	10/10 (100)	3/3 (100)	13/13 (100)	6/10 (60)	3/3 (100)	9/13 (69.2)	6/10 (60)	3/3 (100)	9/13 (69.2)
**Chicken livers**	46/46 (100)	67/67 (100)	113/113 (100)	46/46 (100)	67/67 (100)	113/113 (100)	5/46 (10.9)	64/67 (95.5)	69/113 (61.1)	5/46 (10.9)	64/67 (95.5)	69/113 (61.1)
**Pork**	0/0 (0)	2/2 (100)	2/2 (100)	0/0 (0)	2/2 (100)	2/2 (100)	0/0 (0)	2/2 (100)	2/2 (100)	0/0 (0)	2/2 (100)	2/2 (100)
**Turkey**	5/5 (100)	2/2 (100)	7/7 (100)	5/5 (100)	2/2 (100)	7/7 (100)	3/5 (60)	1/2 (50)	4/7 (57.1)	3/5 (60)	1/2 (50)	4/7 (57.1)

* np: number of positive isolates, n: total number of isolates screened.

### 3.3. Correlation between Genotype and Arsenic Resistance

**Figure 4:**
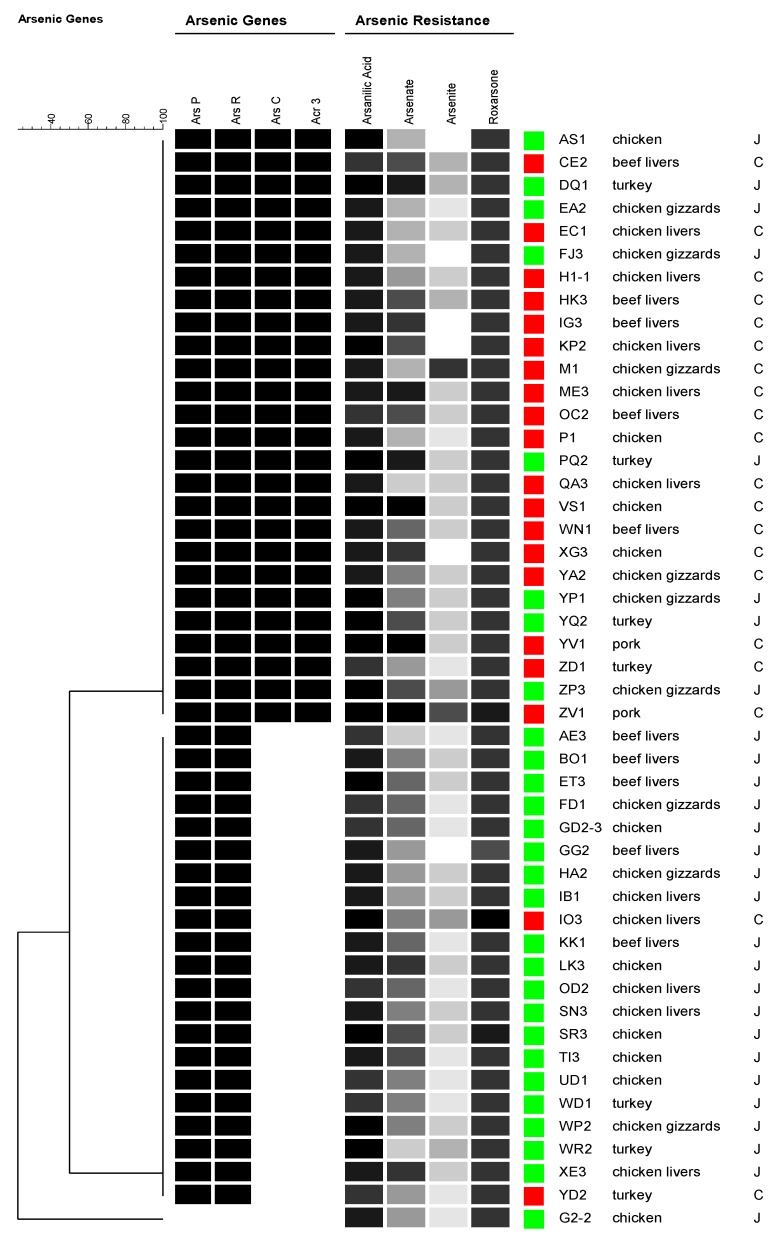
A dendrogram created using the BioNumerics software showing a simple comparison of a 48 representative isolates of the six different meat sources by species showing the type of arsenic resistance gene operon harbored and the anti-arsenic resistance profiles. The intensity of the shaded box is related to the arsenic concentration tolerated by the isolate. The darker the box the higher the concentration tolerated for each arsenic compound. *Campylobacter jejuni* are labeled with red squares and *Campylobacter coli* are labeled with green squares. The meat source is listed by each isolate code as chicken livers, chicken gizzards, chicken, beef livers, turkey, and pork. The four tested anti arsenic compounds are listed on the top of the dendrogram right after the screened arsenic resistance genes.

As shown in Figure 1, arsenic resistance in *Campylobacter* is controlled by a four gene operon. ArsR is actually a negative regulator that binds to the promoter region of the operon and represses the transcription. Arsenite is believed to abolish the binding of *arsR* to the promoter region and transcription will occur [11]. In our study, the presence of *arsC* and *acr3* genes in the large operon did not appear to significantly increase *Campylobacter* resistance to the tested arsenic compound (data not shown). The only noticeable difference was in regards to arsenate resistance where the only isolates that were able to grow at (4,096–8,192 μg/mL) were those harboring the *arsC* and *acr3* genes regardless of the *Campylobacter* species. So, having four genes did not seem to increase the resistance to arsenic compounds when compared to the isolates with two genes, except for allowing some isolates to resist higher arsenate concentrations. Having the *arsC* (an arsenate reductase) in the larger arsenic resistance operon might have a role in conferring such a higher resistance to arsenate. This can also explain our results above where the only noticeable difference between the two tested *Campylobacter* species was that 15.5% of the *Campylobacter coli* isolates were resistant to higher concentrations of arsenate (2,048–80,192 μg/mL) where only 8.5% of *Campylobacter jejuni* isolates were resistant to the same concentration range. The higher number of *Campylobacter coli* isolates resistance to high arsenate concentrations might be due to the fact that the majority of *Campylobacter coli* isolates harbor the larger arsenic resistance operon that carry the arsenate reductase gene (*arsC*). Our finding is consistent with a previous study where they noticed that the presence of *arsC* and *acr3* caused an increase in the resistance of the *Campylobacter jejuni* strains to arsenate and arsenite, even though they tested only 27 isolates in their study [11]. As shown in Figure 4, it is clear from the dendrogram that the presence of the four gene operon did not significantly increase arsenic resistance with the exception of arsenate where some isolates showed resistance to higher concentrations to this compound indicated by darker boxes in the figure which support our discussion above regarding resistance to arsenate. Most of the turkey *Campylobacter jejuni* strains harboring the large operon showed higher resistance to arsenate (Figure 4). The presence of *arsB* did not seem to increase resistance to any of the four tested arsenic compound by *Campylobacter jejuni* isolates in our study (Data not shown) since it was present in 98% of those isolates despite their slight resistance variation. The complete absence of *arsB* from any of the screened *Campylobacter coli* isolates is very interesting. To rule out the possibility of using a non-specific primer, the published DNA sequence for *arsB* from *Campylobacter jejuni* was blasted against the Genbank database including microbial genomes and no homology was found to this gene in any *Campylobacter coli* genome sequence. This can encourage us to suggest that there are still other possible unexplored mechanisms in conferring arsenic resistance to *Campylobacter*. A possible role of the CmeABC efflux pump in this regard is currently under investigation in our laboratory.

## 4. Conclusions

In conclusion, arsenic resistance is prevalent in *Campylobacter jejuni* and *Campylobacter coli* isolated from retail meats and a larger resistance operon is more prevalent in the *Campylobacter coli* strains. The presence of the four gene operon did not significantly increase arsenic resistance compared to the two gene operon with the exception of conferring resistance to higher concentrations of arsenate to some *Campylobacter* isolates. The arsenic resistance gene, *arsB* was prevalent in 98% of the *Campylobacter jejuni* isolates but was completely absent in the *Campylobacter coli* ones. The higher resistance of *Campylobacter* isolates to arsenic compounds in this study is alarming and warrants the FDA to revisit the tolerance levels set for the use of roxarsone in feed. To our knowledge, this is the first study to determine arsenic resistance and the prevalence of arsenic resistance genes in such a large number of *Campylobacter* isolates.
